# Neonatal Multisystem Inflammatory Syndrome (MIS-N) Associated with Prenatal Maternal SARS-CoV-2: A Case Series

**DOI:** 10.3390/children8070572

**Published:** 2021-07-02

**Authors:** Ravindra Pawar, Vijay Gavade, Nivedita Patil, Vijay Mali, Amol Girwalkar, Vyankatesh Tarkasband, Sanjog Loya, Amit Chavan, Narendra Nanivadekar, Rahul Shinde, Uday Patil, Satyan Lakshminrusimha

**Affiliations:** 1 Department of Pediatrics, Dr. D Y Patil Medical College Hospital and Research Institute, Kolhapur 416003, MH, India; patilnivedita8@gmail.com (N.P.); dr.vijaymali@gmail.com (V.M.); 2 Masai Children’s Hospital, Kolhapur 416002, MH, India; vijaygavade@gmail.com (V.G.); sanjog877@gmail.com (S.L.); dramit.chavan15@gmail.com (A.C.); drudayspatil@gmail.com (U.P.); 3 NICE Advanced Neonatal Care Centre and Children’s Clinic, Kolhapur 416008, MH, India; 4 Ratna NICU, Kolhapur 416003, MH, India; amolgirwalkar@gmail.com; 5 Department of Pediatrics, Apple Saraswati Multispeciality Hospital, Kolhapur 416003, MH, India; drt_vyankatesh@yahoo.co.in; 6 Niramay Pediatric Nursing Home and Eye Care Centre, Kolhapur 416001, MH, India; narendrananivadekar@gmail.com; 7 Samarth Nursing Home, Kolhapur 416002, MH, India; drrahulshinde@gmail.com; 8 UC Davis Children’s Hospital, Sacramento, CA 95817, USA; slakshmi@ucdavis.edu

**Keywords:** neonate, multisystem inflammatory syndrome in children (MIS-C), anti SARS-CoV-2 antibodies, COVID-19

## Abstract

Multisystem inflammatory syndrome in children (MIS-C) is a post-infectious immune-mediated condition, seen 3–5 weeks after COVID-19. Maternal SARS-CoV-2 may potentially cause a similar hyperinflammatory syndrome in neonates due to transplacental transfer of antibodies. We reviewed the perinatal history, clinical features, and outcomes of 20 neonates with features consistent with MIS-C related to maternal SARS-CoV-2 in Kolhapur, India, from 1 September 2020 to 30 April 2021. Anti-SARS-CoV-2 IgG and IgM antibodies were tested in all neonates. Fifteen singletons and five twins born to eighteen mothers with a history of COVID-19 disease or exposure during pregnancy presented with features consistent with MIS-C during the first 5 days after birth. Nineteen were positive for anti-SARS-CoV-2 IgG and all were negative for IgM antibodies. All mothers were asymptomatic and therefore not tested by RTPCR-SARS-CoV-2 at delivery. Eighteen neonates (90%) had cardiac involvement with prolonged QTc, 2:1 AV block, cardiogenic shock, or coronary dilatation. Other findings included respiratory failure (40%), fever (10%), feeding intolerance (30%), melena (10%), and renal failure (5%). All infants had elevated inflammatory biomarkers and received steroids and IVIG. Two infants died. We speculate that maternal SARS-CoV-2 and transplacental antibodies cause multisystem inflammatory syndrome in neonates (MIS-N). Immunomodulation may be beneficial in some cases, but further studies are needed.

## 1. Introduction

COVID-19, caused by SARS-CoV-2, is a global public health crisis with a large recent surge in India. As of 24 June 2021, 179 million individuals were infected worldwide, with India contributing to half of all new daily cases in April–May 2021 [1]. Initial studies showed that children were spared of severe COVID-19 [2,3,4]. However, recently case reports of children experiencing a potentially life threatening pediatric inflammatory multisystem syndrome (PIMS)—also called multisystem inflammatory syndrome in children (MIS-C)—have been described [5,6,7].

MIS-C is a new disease in children, the exact mechanism of which is still unclear. It is thought to be due to immune dysregulation following exposure to SARS CoV-2 [8]. It usually presents as fever and multiorgan involvement, with blood investigations showing increased inflammatory markers weeks after exposure to SARS-CoV-2 [5,6,8]. MIS-C has clinical and serological similarities with Kawasaki disease and the severe COVID-19 cytokine storm seen in adults [9]. However, its pathophysiology and immunological response is different, and may be mediated by autoantibodies [10]. More than 80% of children with MIS-C have specific IgM and IgG antibodies against SARS-CoV-2, but only about one-third are positive for SARS-CoV-2 by RTPCR [5,11,12].

Unlike MIS-C, where SARS-CoV-2 infection and multisystem inflammation occur in the same subject, a few case reports suggest neonatal multisystem inflammation [13] occurs secondary to maternal SARS-CoV-2 infection [14,15,16,17]. A few weeks after the first wave of COVID-19 in Kolhapur, India, we found an increase in the number of neonates with structurally normal hearts who presented with conduction abnormalities and were born to mothers with a past history of COVID-19. Specifically, these neonates presented with prolonged QTc with 2:1 Atrioventricular (AV) block or thrombosis similar to older children with MIS-C within the first week after birth [18]. We present a case series of 20 neonates with multisystem involvement, hyperinflammatory syndrome and positive anti SARS-CoV-2 IgG antibodies, temporally related to maternal antenatal SARs-CoV-2 exposure. To our knowledge, this is the largest series of MIS-C presenting in the early neonatal period.

## 2. Materials and Methods

Access to chart reviews and publication was approved by the Institutional Ethics Committee (IEC) of the Dr D Y Patil Medical College Hospital and Research Institute, at Dr D Y Patil University, Kolhapur, India. Informed consent was obtained from parents/guardians for using clinical data and photographs. Neonates who met the criteria in Table 1 (with four exceptions, as explained below) and that were admitted to seven NICUs in Kolhapur between 1 September 2020 and 30 April 2021 were included. These criteria were modified from CDC criteria for MIS-C and interim guidance from AAP to accommodate lack of fever in neonates and source of primary infection (mother, instead of the child) [19,20]. Neonates with signs consistent with MIS-C, maternal history of COVID-19, and positive for anti-SARS CoV-2 antibodies were included. However, infants with these symptoms and culture positive sepsis, or proven infective pathology in other organ systems (e.g., meningitis, urinary tract infection, etc.) were excluded. Infants with low Apgar scores (≤3 at 5 min) and evidence of birth asphyxia were excluded. Preterm infants with findings attributable to early gestation (such as respiratory distress presenting immediately after birth and transient hypotension) were excluded. IgG and IgM against SARS CoV-2 were detected using VIDAS^®^ SARS-COV-2 kits (BioMerieux SA, Marcy-I’Etioile, France), with MINIVIDAS using ELFA: enzyme linked fluorescent assay. Data are presented as median (range) or number (%).

**Figure 1:**
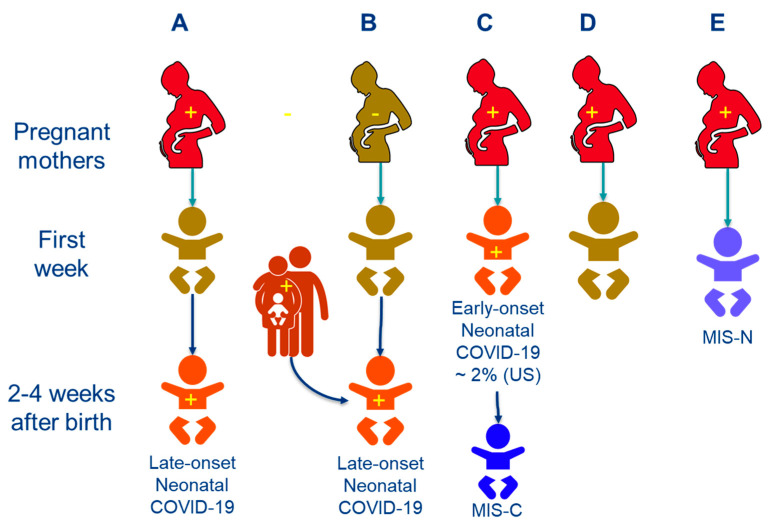
Various presentations of SARS-CoV-2 infection and its sequences in the neonatal period. Red colored subjects with a ‘+’ sign indicate COVID-19 positive patients. Pregnant mother A has COVID-19 and her baby is negative at birth but contracts late-onset COVID-19 due to transmission from the mother. Pregnant mother B has no COVID-19 but her neonate develops late-onset neonatal infection due to exposure to a family member 2–4 weeks after birth. Pregnant mother C is COVID-19 positive during the perinatal period and transmits the virus to her offspring during birth leading to early-onset infection in the neonate. This baby can potentially develop MIS-C 2–4 weeks later (a rare occurrence) [16]. Pregnant mother D has COVID-19 during pregnancy but the neonate remains healthy. Pregnant mother E has COVID-19 disease or exposure to SARS-COV-2 during pregnancy and the baby develops multisystem inflammation secondary to passive transfer of antibodies leading to MIS-N (multisystem inflammatory syndrome in neonates) [14].

We differentiated neonates presenting with multisystem inflammatory syndrome in the first week after birth secondary to possible maternal COVID-19 infection (labeled in this article as MIS-N), from neonates who had early onset neonatal COVID-19 or late-onset neonatal COVID-19 and subsequently present with multisystem inflammation during 2–4 weeks after birth (labeled in this article as MIS-C) (Figure 1). In patients with MIS-C, multisystem inflammation was secondary to prior COVID-19 in the same subject. However, in MIS-N, multisystem inflammation in the neonate was secondary to COVID-19 in the mother with passive transmission of antibodies.

## 3. Results

Clinical characteristics of 20 neonates are shown in Table 2. Individual patient characteristics are shown in Table 3. Three infants (# 17, 18 and 19 in Table 3) had IgG anti SARS-CoV-2 levels below the cut-off but were included because of maternal history and typical presentation (AV block or dilated coronaries). Case # 20 only had a cardiac thrombus without other organ involvement but was included due to maternal history, high IgG levels, elevated inflammatory markers, and lack of other explanation for the thrombus.

### 3.1. Maternal Features

Of the 18 mothers (three with twin pregnancy), seven (38.8%) were symptomatic for COVID-19 during pregnancy, three (16.6%) were asymptomatic but RT-PCR positive for COVID-19, and eleven (61.1%) were asymptomatic but had history of close contact with COVID-19 cases (usually a confirmed case in the family). Fifteen mothers (83.3%) were symptomatic or had contact during the last trimester of pregnancy, (five (27.7%) within the last 4 weeks before delivery), two (11.1%) during second trimester and one (5.5%) in the first trimester of pregnancy. None of them had symptomatic COVID-19 or febrile illness during admission for delivery, and none were tested for COVID -19 RT-PCR during the admission for delivery. Five mothers (27.7%) had an antenatal ultrasound scan showing fetoplacental compromise (reduced flow in uterine artery or umbilical artery and/or diastolic notch, diastolic flow reversal, fetal ascites, pericardial and pleural effusion). Mothers whose infants presented with cardiac conduction abnormalities were tested for lupus antibodies and were negative.

### 3.2. Resuscitation at Birth and Post-Resuscitation Period

Two neonates did not cry immediately after birth and two had significant respiratory distress in the delivery room. These four (20%) neonates required positive pressure ventilation (PPV) and subsequently required conventional mechanical ventilation on the day of birth. Sixteen (80%) neonates did not require any PPV in the delivery room. However, three of these infants required respiratory support (invasive mechanical ventilation or CPAP) on the day of birth in the NICU. 

### 3.3. Clinical Presentation

**Figure 2:**
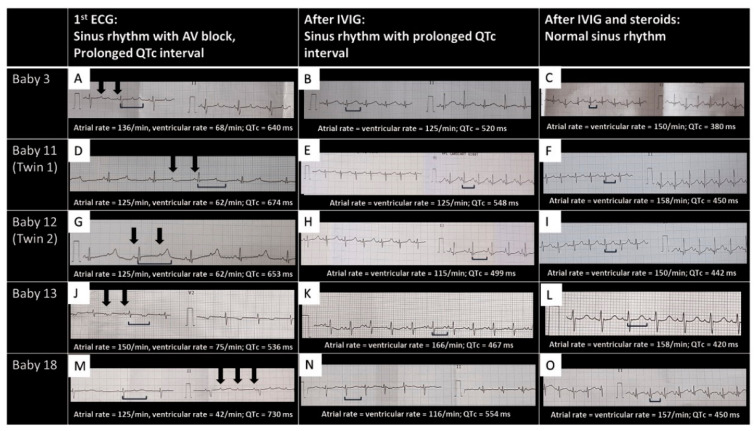
Representative EKGs of neonates presenting with bradycardia. Baby number sequence is the same as in Table 3. The first column showing EKGs at presentation (**A**,**D**,**G**,**J**,**M**), with sinus rhythm, prolonged QT interval and atrio-ventricular block. Middle column showing sinus rhythm and prolonged QT interval (**B**,**E**,**H**,**K**,**N**). The last column showing sinus rhythm with normal QT interval (**C**,**F**,**I**,**L**,**O**). Black arrows = atrial beats; horizontal square bracket = QT interval; QTc = corrected QT interval; ms = milliseconds. QTc values in the figure are derived by the formula QTc = QT/√RR.

**Figure 3:**
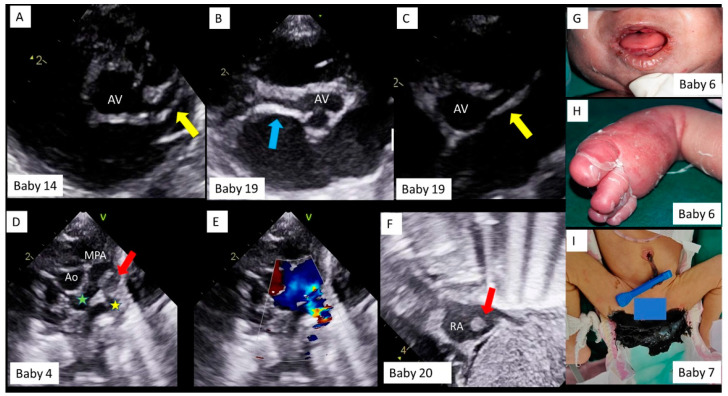
Echocardiography and clinical findings in neonates with MIS-C. Baby number sequence is the same as in Table 3. Transthoracic echocardiography, parasternal short axis view in Baby #14 (**A**) and Baby #19 (**B**,**C**). The left main and left anterior descending coronary artery (yellow arrow) and the right coronary artery (blue arrow) are significantly dilated. AV = aortic valve. Transthoracic echocardiography and color doppler, parasternal short axis view in Baby #4 (**D**), showing aorta (Ao) and main pulmonary artery (MPA) bifurcation, with a large thrombus (red arrow) obstructing the left pulmonary artery (yellow star) origin and causing flow turbulence on color doppler (**E**), but normal flows across right pulmonary artery (green star). Transthoracic echocardiography subcostal bi-caval view in Baby # 20 (**F**), showing a thrombus (red arrow) in right atrium (RA). Baby #6, showing oral and muco-cutaneous lesions (**G**) and, pedal edema and skin peeling (**H**) and Baby #7 with black, tarry stools (melena, **I**).

The most common presentation involved the cardiovascular system (Table 3). Eleven had rhythm disorders, of which nine presented with prolonged QTc interval with 2:1 AV block (Figure 2A,D,G,J,M). With immunomodulatory therapy with methylprednisolone and intravenous immunoglobulin (IVIG), 2:1 AV block disappeared first (Figure 2B,E,H,K,N), followed by normalization of QTc (C, F, I, L, O), in all of the nine neonates. One neonate had an episode of supraventricular tachycardia (SVT), requiring a short course of beta blockers, and one infant had bradycardia with tall, peaked T waves and broad QRS due to hyperkalemia secondary to acute renal failure. Shock with or without cardiac dysfunction on echocardiography was seen in five neonates. Two neonates had significant coronary dilatation on day one of life (Figure 3A–C). One neonate had a thrombus almost completely occluding the left pulmonary artery (LPA) (Figure 3D), requiring systemic thrombolysis with Alteplase (t-PA, 3 doses), and low molecular weight heparin (LMWH) for six weeks. One neonate had an intracardiac thrombus at the inferior vena cava–right atrial junction (Figure 3E), which partly resolved at discharge, after LMWH therapy. 

Eleven neonates required either mechanical ventilation (n = 8) or CPAP (n = 3), for respiratory distress syndrome associated with prematurity, shock, or respiratory depression. Two neonates presented with fever on day one of life. Two neonates did not cry immediately after birth but had Apgar scores >3 by 5 min of age. One infant presented with convulsions on day 4 and was admitted on day 6 with multiorgan failure leading to death. 

Feeding intolerance and gastric aspirates were seen in 6 neonates, of which two had brownish gastric aspirates. Two had lower gastrointestinal bleeding, of which one had tarry stools (melena) (Figure 3I) and one had blood in stools on day 8 of life (with a normal coagulation profile). 

Anti-SARS-CoV-2 IgM antibodies were negative in all the neonates, and IgG antibodies (cut-off-index (COI) ≥1 considered reactive) were positive (COI value > 1) in 17 (85%) neonates. Two (10%) had levels below positive cut-off, and one (5%) had no detectable levels. RTPCR for SARS-CoV-2 was not done in any of the neonates as the Indian Academy of Pediatrics Guidelines recommend this test after birth if mothers are symptomatic, or tested positive within 14 days before birth, or if there is history of contact with COVID-19 positive persons in the postnatal period. [21]

To summarize, we present a case series of 20 neonates born to mothers with a history of SARS-CoV-2 infection or exposure to COVID-19 patients. The majority of infants were late preterm, with equal sex distribution and presented with cardiac (90%), respiratory (55%) or gastrointestinal (30%) signs with elevated inflammatory markers and positive IgG SARS-CoV-2 titers. These infants were managed with supportive therapy, methylprednisolone, IVIG and was associated with a 10% mortality. Our protocol for diagnosis and management of MIS-N is shown in Table 4.

## 4. Discussion

We present a case series of neonates born to mothers with a history of SARS-CoV-2 infection or exposure to a COVID-19 patient during pregnancy and presenting with features that cannot be explained by other causes. Whether these findings are unrelated to maternal COVID-19 or due to an inflammatory process induced by the transplacental passage of antibodies directed against autoantigens is not clear. However, the unusually high frequency of findings such as atrioventricular conduction abnormalities, resembling cardiac findings in older children with MIS-C [18], and response to immunomodulatory therapy with intravenous immunoglobulin (IVIG) and steroids suggests that “multisystem inflammatory syndrome in the neonate (MIS-N)” deserves further study [26]. We present this case series to increase awareness of this possibility amongst all care providers, especially obstetricians, pediatricians, pediatric cardiologists, and neonatologists.

We speculate that maternal infection with SARS CoV-2 results in development of protective IgG antibodies against spike protein of the virus (similar to a response following vaccination) [27]. These antibodies cross the placenta (with IgA versions in breastmilk) to provide passive immunity to the newborn [27]. In some genetically susceptible children, autoantibodies triggered by SARS CoV-2 infection may bind to receptors in neutrophils and macrophages causing activation and secretion of pro-inflammatory cytokines that results in development of MIS-C [9,28]. Children with MIS-C have higher SARS-CoV-2 IgG titers than those with severe COVID-19 [29], however, this trend is transient in MIS-C [30]. We speculate that the spike protein IgG antibodies are protective innocent bystanders, are a marker of prior infection and do not have a pathogenic role in MIS-C. On the other hand, autoantibodies against endothelial, gastrointestinal, and immune cells are also produced and may potentially play a role in MIS-C [31]. Patients with MIS-C have high levels of certain antibodies against autoantigens (anti-SSB, anti-Jo-1), lending credence to the hypothesis that MIS-C is mediated by a persistent autoimmune response to the original infection [31]. As such, and analogous to neonatal lupus, where anti-SSA and anti-SSB antibodies cross the placenta to cause manifestations such as rash and congenital heart block in newborns, it is plausible that similar antibodies against autoantigens crossed the placenta after a SARS CoV-2 infection and initiated MIS-N disease in these neonates. In our case series, atrioventricular conduction abnormalities were common (Figure 2) potentially secondary to transplacental transfer of similar antibodies.

We would like to differentiate MIS-C in the neonatal period due to early-onset SARS-CoV-2 infection in the neonate from “MIS-N” where the infection occurs in the mother and the neonates present early as shown in this case series (Figure 1). We acknowledge that the CDC has not labelled or described this condition and nomenclature may change in the future.

That maternal antibodies pass transplacentally is a known fact, and maternal infection with SARS-CoV-2 is no different. Multiple studies have reported the transplacental transfer of anti-SARS-CoV-2 IgG antibodies to neonates [32,33,34]. The majority (87%) infants born to seropositive mothers had detectable IgG antibody at birth, transfer ratios were more than 1.0, and there was a positive correlation between maternal and infant antibody titers, regardless of the presence of symptoms in the mother or the severity of disease [33].

None of the mothers in our case series had received vaccination against COVID-19 (vaccines were only administered to >45 years age strata in India during the study period). Although COVID-19 vaccines were not tested in pregnant mothers, many pregnant health care workers have received the Pfizer and Moderna vaccines in the US [35]. These mothers have a robust IgG and IgA response in their sera and breast milk respectively [27]. Umbilical cord sera were positive for IgG antibodies. We speculate that these vaccine induced antibodies against SARS CoV-2 spike protein are protective and do not pose a risk of MIS-C in babies because they are not directed towards autoantigens. Approximately 4500 pregnant mothers have registered in the V-safe COVID-19 vaccine pregnancy registry. Limited data from this registry have not reported any neonatal deaths to date [9,36].

The majority of infants in our case series were delivered at late preterm gestation. The NICHD Maternal Fetal Medicine Units (MFMU) network has reported a higher incidence of preterm labor and delivery in symptomatic pregnant mothers with COVID-19 (severe symptoms–42%; mild to moderate symptoms—15%; asymptomatic—12%) [37]. Therapy for MIS-N is mainly supportive. All patients in our case series received immunomodulatory therapies (intravenous immunoglobulin-IVIG and steroids), anti-platelet agents (aspirin), and anticoagulants (unfractionated heparin or LMWH). Further studies are required to evaluate the benefits and risks of these therapies in MIS-N [23,25]. While some cases, especially those with cardiac conduction abnormalities responded well to IVIG and steroid therapy, we need randomized trials to evaluate efficacy of these therapies in MIS-C. Overuse of these agents should be avoided. We admit that there was probably overtreatment with steroids, LMWH and IVIG among our patients and many of these patients might have improved without these therapies. More targeted therapy with these agents based on further research is prudent as IVIG use among neonates carries the potential risk of necrotizing enterocolitis [24].

## 5. Conclusions

We conclude that maternal history of SARS-CoV-2 infection or exposure to COVID-19 may potentially be associated with multisystem inflammation, thrombosis, and AV conduction abnormalities in the early neonatal period. However, neonatal MIS-C and MIS-N are relatively rare. More common causes for cardiac dysfunction and elevated troponin or BNP such as perinatal asphyxia and sepsis should be considered. Based on our case series, we recommend that among neonatal patients born to mothers with a history of COVID-19, neonatal MIS-C or MIS-N be considered in the differential diagnosis to explain unusual signs of multisystem inflammation, after excluding common causes.

## Figures and Tables

**Table 1:** Proposed inclusion criteria for neonatal multisystem inflammatory syndrome (MIS-N) secondary to maternal SARS CoV-2 exposure or infection.

(1) A neonate aged <28 days at the time of presentation (2) Laboratory or epidemiologic evidence of SARS-CoV-2 infection in the mother Positive SARS-CoV-2 testing by RT-PCR, serology (IgG or IgM), or antigen during pregnancy Symptoms consistent with SARS CoV-2 infection during pregnancy COVID-19 exposure with confirmed SARS CoV-2 infection during pregnancy Serological evidence (positive IgG specific to SARS CoV-2 but not IgM) in the neonate (3) Clinical criteria: Severe illness necessitating hospitalization AND Two or more organ systems affected [i.e., cardiac, renal, respiratory, hematologic, gastrointestinal, dermatologic, neurological, temperature instability (fever or hypothermia)] OR Cardiac AV conduction abnormalities OR coronary dilation or aneurysms (without involvement of a second organ system) (4) Laboratory evidence of inflammation One or more of the following: an elevated CRP, ESR, fibrinogen, procalcitonin, D-dimer, ferritin, LDH, or IL-6; elevated neutrophils or reduced lymphocytes; low albumin (5) No alternative diagnosis (such as birth asphyxia–cord pH ≤ 7.0 and Apgar score ≤ 3 at 5 min; viral or bacterial sepsis–confirmed blood culture; maternal lupus resulting in neonatal AV conduction abnormalities; presence of these findings indicating an alternate diagnosis excludes MIS-N).

**Table 2:** Characteristics of patients with suspected MIS-N.

Characteristic	Median (Range) or Number (%)	Comments
Characteristic	Median (Range) or Number (%)	Comments
Maternal age	26.5 years (20–34 years)	
Maternal symptoms (n = 18):		
Asymptomatic Symptomatic Trimester when positive or had exposure	11 (61.2%) 7 (38.8) First–1 (5.5%), Second–2 (11.1%), Third–15 (83.3%)	
Mode of delivery–Cesarean	7 (38.8%)	
Gestational age at birth (n = 20)	34 weeks (27–38 weeks)	Term (≥37 weeks)–3 (15%) Late preterm (34–36 weeks)–13 (65%) <33 weeks–4 (20%)
Birth weight (kg)	2.15 (1–4)	
Sex	Male (10), Female (10)	
Multiplicity	Singleton–15 Twins–5	
Neonate–age at presentation	Day 2 (day 1 to day 5)	Day 1 (<24 h of birth)–7 (35%)
Organ system involvement		
• Cardiac • Hematologic/thrombosis • Respiratory • Gastrointestinal • Neurological • Cutaneous • Renal • Fever/temperature instability	18 (90%) 2 (10%)-thrombosis; 2 (10%)-GI bleed 11 (55%)-requiring ventilator/CPAP 6 (30%) 2 (10%) 1 (5%) 1 (5%) 2 (10%)	
Investigations:		
• CRP • Procalcitonin • D-dimer • LDH • NT-Pro BNP	24 (9–62) 2.05 (1.3–51) 5932 (2820–12,000) 1315 (793–6424) 24,300 (7361-> 30,000)	Normal values 0-6 mg/L <0.5 ng/mL <2700 ng/mL 290-775 U/L <11,987 pg/mL for 0-2 days,
		<5918 pg/mL for 3-11 days
• Blood culture	No growth in all infants	No growth
Cardiac findings		
• Arrhythmia • Dilated coronaries • Intracardiac thrombus • Shock/ cardiac dysfunction	11 (44%) 2 (12%) 2 (8%) 5 (20%)	One thrombus in LPA, one in RA
Infant’s serology		
• IgM SARS CoV-2 • IgG SARS CoV-2	0.0 (0–0) COI 3.49 (0.07–74.39) COI	Cut-off-Index (COI) ≥ 1 is positive, for both IgG and IgM
Therapy		
• Steroids • IVIG • LMWH • Inotropes	20 (100%) 20 (100%) 14 (70%) 12 (60%)	IV Methylprednisolone 2 mg/kg/day 1–2 g/kg 1.5 mg/kg/dose, twice a day IV Milrinone, Adrenaline, Dobutamine, Dopamine
Outcome		
• Mortality	2 (10%)	One due to necrotizing enterocolitis One due to Multiorgan dysfunction

**Table 3:** Clinical features, treatments, and outcomes of suspected patients with Neonatal Inflammatory Multisystem Syndrome (MIS-C) associated with SARS-CoV-2 infection.

Subject Number	Age at Presentation/Sex/Weight/Gestation	Maternal COVID-19 Status	Neonatal Serology (All Were IgM-ve)	Lab Studies (Values)	Clinical Features	Treatment	Outcome
1 *	Day 1/F 4 kg 38 weeks	asymptomatic, RTPCR +ve 3 weeks before delivery	IgG +ve on day 1	Elevated CRP (14), PCT (1.3), Ferritin (1500), D Dimer (5088).	Fever on day 1, hypotension; echo-LV dysfunction	Inotropes Steroids IVIG	Discharged on day 13
2 *	Day 1/M 2.02 kg 35 weeks	asymptomatic, COVID-19 contact 8 weeks before delivery,	IgG +ve on day 2	Elevated CRP (9), D-Dimer (5100), Ferritin (393), LDH (1183), NT Pro BNP (>30,000)	Antenatal scan showing fetoplacental compromise; shock on day 1, Echo—mild LV dysfunction and bilateral pleural effusions	LMWH Steroids IVIG	Discharged on day 14
3 *	Day 4/F 2 kg 33 weeks	asymptomatic COVID-19 contact 6 weeks before delivery, IgG +ve	IgG +ve on day 6	Elevated CRP (10), d-dimer (3020), Ferritin (407)	RDS, severe bradycardia with prolonged QTc and 2:1 AVB from day 4 of life (Figure 2A,B)	Surfactant MV, Steroids IVIG	Sinus rhythm at discharge on day 16 (Figure 2C)
4 *	Day 1/M 2 kg 36 weeks	asymptomatic COVID-19 contact 6 weeks before delivery,	IgG +ve on day 1	Elevated CRP (12), D Dimer (6848), NT Pro BNP (>25,000), LDH (1158)	Antenatal scan showing dilated RA/RV, pericardial and pleural effusions and ascites; Respiratory distress, PPV at birth, shock; echo–dilated hypertrophied RV with dysfunction, moderate TR, large thrombus at LPA origin on day 3 (Figure 3D,E)	LMWH, Alteplase, Inotropes, MV, IVIG, Steroids	Discharged on day 19; LMWH and Aspirin x 6 weeks, complete resolution of thrombus at 8 weeks echo
5 *	Day 3/M 3.5 kg 38 weeks	Febrile illness at 7 months of gestation	IgG +ve on day 5	Elevated CRP (60.2), PCT (2.1), D Dimer (6483), Ferritin (878), LDH (793), leucocytosis (18,600).	Grunting, tachypnea, and lethargy, feeding intolerance, intermittent bradycardia, hypotension	Inotropes, MV, Steroids, IVIG	Discharged on day 13
6 *	Day 2/M 2.3 kg 34 weeks	Febrile illness 2 weeks before delivery	IgG + ve on day 12	Elevated CRP (24), d dimer (4200), thrombocytopenia (39 × 10^9^/L)	feeding intolerance, decreased activity from day 2, brown gastric aspirates on day 4, treated like NEC, bleeding continued with rash, pedal edema, oral and skin lesions, skin peeling (Figure 3G,H)	CPAP, Inotropes, LMWH, Steroids, IVIG	Discharged on day 38
7 *	Day 3/F 1.4 kg 34 weeks	Asymptomatic RTPCR +ve, 5th month of gestation	IgG +ve on day 5	Elevated CRP (50), D Dimer (5100), normal coagulation profile	Antenatal scan showing fetoplacental compromise; LBW. Brownish gastric aspirates from day 3, frank malena (Figure 3I) from day 6, episodes of SVT from day 8; Echo- bilateral pleural and pericardial effusion.	Beta- blockers, Steroids, IVIG	Discharged on day 20
8 * **2nd of twins †**	Day 2/M 1.9 kg 32 weeks	Asymptomatic RTPCR positive at 3rd month of gestation	IgG +ve on day 1	Elevated CRP (43), IL-6 (116), D Dimer (6600)	distress at birth, bradycardia with prolonged QTc and 2:1 AVB on day 2 of life	MV and CPAP, inotropes, Steroids, IVIG	Sinus rhythm at discharge on day 23
9 * **Twin 1**	Day2/F 1.9 kg 33 weeks	Asymptomatic COVID-19 contact 8 weeks before delivery	IgG +ve on day 4	Elevated CRP (35), D Dimer (10,000)	Antenatal scan showing fetoplacental compromise, bradycardia with prolonged QTc and 2:1 AVB from day 2	IVIG, Steroids, LMWH,	Sinus rhythm at discharge on day 18
10 * **Twin 2**	Day 2/M 1.6 kg 33 weeks	Asymptomatic COVID-19 contact 8 weeks before delivery	IgG +ve on day 5	Elevated D Dimer (10,000), LDH (977)	Antenatal scan showing feto-maternal compromise, feeding intolerance, bradycardia with prolonged QTc and 2:1 AVB from day 2,	IVIG, steroids, LMWH	Sinus rhythm at discharge on day 18
11 * **Twin 1**	Day 4/F 2.05kg 34 weeks	Febrile illness 3 weeks before delivery–IgG level below cutoff	IgG +ve on day 4	Elevated PCT (1.8), D Dimer (4840), NT Pro BNP (> 25,000)	bradycardia with prolonged QTc and 2:1 AVB on day 4 (Figure 2D,E)	IVIG, steroids, LMWH, inotropes, CPAP	Sinus rhythm at discharge on day 11 (Figure 2F)
12 * **Twin 2**	Day 4/M 2.1 kg 34 weeks	Febrile illness 3 weeks before delivery–IgG level below cutoff	IgG +ve on day 4	Elevated PCT (1.4), D Dimer (5932)	bradycardia with prolonged QTc and 2:1 AVB on day 4 (Figure 2G,H)	IVIG, steroids, LMWH, inotropes, CPAP	Sinus rhythm at discharge on day 11 (Figure 2I)
13 *	Day 3/F 1 kg 27 weeks	Asymptomatic COVID-19 contact 8 weeks before delivery	IgG +ve on day	Elevated PCT (51), D Dimer (10,000), LDH (6424), NT Pro BNP (25,000)	Extreme PT, Extreme LBW, bradycardia with prolonged QTc and 2:1 AVB with 2:1 AV block on day 4 (Figure 2J,K); sinus rhythm on day 7 (Figure 2L)	IVIG, Steroids, LMWH, MV	day 9 abdominal distension, NEC → death on day 11
14 *	Day 2/M 2.4 kg 36 weeks	Asymptomatic COVID-19 contact 10 weeks before delivery, IgG +ve	IgG +ve on day 6	Elevated CRP (11), D Dimer (4700), LDH (2143)	not accepting feeds on day2, Cardiomegaly on X-ray chest, cardiogenic shock on day 5, echo (Figure 3A)-dilated coronaries ^#^ (LMCA Z score = + 4.2, RCA Z score = +4.9) severe TR, mild MR, ASD, PDA, Severe PAH,	IVIG, steroids, LMWH, Inotropes, PPV, Aspirin	Discharged on day 14; Coronaries normalized at discharge, Tab Aspirin x 6 weeks
15 *	Day 4/M 2 kg 36 weeks	Asymptomatic COVID-19 contact 4 weeks before delivery	IgG +ve on day 6	Elevated CRP (18), BUN (99.2), serum Creatinine (1.9), NT Pro BNP (14,500), Potassium (6.9 mEq/L)	Admitted on day 6, Seizures, shock, bradycardia, acute renal failure, hyperkalemia, Echo-small ASD, dilated all four chambers, mild LV dysfunction	IVIG, Steroids, MV, Inotropes, Peritoneal dialysis,	Death on day 8-Multi-organ dysfunction
16 *	Day 1/F 2 kg 36 weeks	Asymptomatic COVID-19 contact 4 weeks before delivery, IgG +ve	IgG +ve on day 1	Elevated CRP (62), PCT (2.4), D Dimer (9734), NT Pro BNP (7361).	Fever on day 1, feeding intolerance, vomiting, tachypnea, desaturation on day 2	IVIG, Steroids	Discharged on day 10
**17**	Day 2/F 1.5 kg 32 weeks	Asymptomatic COVID-19 contact 10 weeks before delivery	IgG -ve	Elevated CRP (18), D Dimer (12,000)	RDS, bradycardia with prolonged QTc and 2:1 AVB on day 3	IVIG, steroids, inotropes, CPAP	Sinus rhythm at discharge on day 13
**18**	Day 2/F 1.5 kg 32 weeks	Febrile illness 8 weeks before delivery	IgG below cut-off level	Elevated CRP (25), D Dimer (10,000), NT Pro BNP (23,700)	bradycardia with prolonged QTc and 2:1 AVB on day 2 (Figure 2M,N)	IVIG, steroids	Sinus rhythm at discharge on day 14 (Figure 2O)
**19**	Day 1/M 1.9 kg 34 weeks	Febrile illness 6 weeks before delivery; IgG below cutoff levels, IgM -ve	IgG below cutoff levels	Elevated D Dimer (2820), LDH (2661), NT Pro BNP (>25,000), thrombocytopenia (93 × 10^9^/L)	Antenatal scan showing pleural, pericardial effusions, and ascites; not cried after birth; LBW, pitting edema over chest wall, hepatomegaly, tachypnea, crepitations; Echo (Figure 3C)—dilated coronaries ^#^, (LMCA Z score = +2.7, LAD Z score = +2.7, RCA Z score = +2), large PDA, mild TR and MR, normal function (on inotropes), bilateral moderate pleural effusion;	IVIG, Steroids, LMWH, Inotropes, Lasix, MV, Aspirin	Discharged on day 15; Coronaries normal, Aspirin x 6 weeks.
**20**	Day 1/ F 2.7 kg 38 weeks	Febrile illness 6 weeks before delivery, IgG +ve	IgG +ve on day 2	Elevated CRP (53), D Dimer (3942), LDH (804), NT Pro BNP (17,018)	Not cried after birth, mottling and poor peripheral pulsations, hypotension; Echo (Figure 2F)–day 4-intracardiac thrombus in RA, normal LV function	PPV, surfactant, Inotropes, IVIG, Steroids, LMWH, MV	Discharged on day 24; LMWH x 6 weeks, thrombus decreased in size at 4 weeks echo

Normal ranges and units for lab values: CRP 0-6 mg/L; D-Dimer < 2740 ng/mL (0-3 days of life); NT Pro-BNP < 11,987 pg/mL for 0–2 days, <5918 pg/mL for 3–11 days; procalcitonin <0.5 ng/mL; LDH 290-775 U/L (for 0-4 days of life); IL-6 < 7 pg/mL; Ferritin = 25–200 ng/mL; BUN 2–19 mg/dL; serum creatinine 0.3–1 mg/dL; patients with (*) met the inclusion criteria mentioned in Table 1. Patients 17–19 did not have a positive IgG SARS CoV-2 level above the laboratory cut-off–however, patients had EKG consistent with AV block; patient 20 had delayed cry and might have had perinatal depression but had Apgar scores > 3 by 5 min but an unexplained intracardiac thrombus in the right atrium. † Twin A was positive for IgG SARS CoV-2 but other clinical features were consistent with prematurity. # Z scores for coronary diameter were calculated based on Kobayashi et al. [22]. Abbreviations: -ve = negative; +ve = positive; M = male, F = female; ASD = atrial septal defect; AVB = atrioventricular block; BUN = blood urea nitrogen; CKMB = creatinine kinase myocardial band; CNS = central nervous system; COVID-19 = corona virus disease 2019; CPAP = continuous positive airway pressure; CRP = C-reactive protein; IgG = immunoglobulin G, IgM = immunoglobulin M, IL-6 = interleukin-6; IVIG = intravenous immunoglobulin; LAD = left anterior descending coronary artery; LBW = low birth weight, LDH = lactate dehydrogenase; LMCA = left main coronary artery; LMWH = low molecular weight heparin; LPA = left pulmonary artery; LV = left ventricle; MR = mitral regurgitation; MV = mechanical ventilation; NEC = necrotizing enterocolitis; NT Pro BNP = N-terminal pro–B-type natriuretic peptide; PAH = pulmonary artery hypertension; PCT = procalcitonin; PDA = patent ductus arteriosus; PPHN = persistent pulmonary hypertension of the newborn; PPV = positive pressure ventilation; PT = preterm; QTc = corrected QT interval; RA and RV = right atrium and ventricle; RCA = right coronary artery; RT- PCR = reverse transcription-polymerase chain reaction; SVT = supraventricular tachycardia; TR = tricuspid regurgitation.

**Table 4:** Protocol for laboratory investigations and management of MIS-N.

**Laboratory investigations** need to be titrated based on clinical presentation. [9] 1. Initial laboratory evaluation (suspected cases without cardiac involvement) Complete Blood Count (CBC) with differential Inflammatory markers: ESR, CRP, Procalcitonin Urinalysis Blood culture Imaging as clinically indicated (respiratory or gastrointestinal signs): Chest X-ray Abdominal X-ray or ultrasound if concerning physical findings. 2. If initial labs concerning for MIS-C, or cases with cardiac involvement without alternate explanation (ESR > = 40 mm or CRP > =5mg/dL in addition to 1 of the following: lymphopenia with absolute lymphocyte count <1000/mm^3^, platelets <150,000/mm^3^, albumin < = 3g/dL, hyponatremia Na < 135 mEq/L), consider the following evaluation: Cardiac markers: troponin T/I and BNP or NT-Pro-BNP Twelve-lead electrocardiogram (EKG) Other markers of inflammation: ferritin, LDH, IL-6 Coagulation panel: PT, PTT, D-dimer, fibrinogen Mother and baby’s Serology for SARS-CoV-2 Mother and baby’s SARS-CoV-2 PCR from nasopharyngeal swab Echocardiogram (transthoracic)–may be done in the presence of hypotension/shock or suspicion for cardiac dysfunction; this may aid in the diagnosis of coronary aneurysms
**Management** of neonates with MIS-N is predominantly supportive. 1. Respiratory support to optimize gas exchange and maintain oxygen saturations in the 90–97% range and PaCO_2_ in the 40–50 mmHg will minimize pulmonary vasoconstriction and reduce the risk of PPHN. 2. Fluid resuscitation along with the use of inotropes and vasopressors is often needed to optimize perfusion. 3. Empiric antibiotics as per discretion of the provider may be considered pending blood culture results.
**Specific therapy** for MIS-C includes the use of anticoagulants, steroids, IVIG and anti-inflammatory agents. As shown in the case reports in Table 2 and Table 3, neonates have received treatment with immunomodulatory therapies (IVIG, methylprednisolone, anti-platelet agents (aspirin), and anticoagulants (unfractionated heparin or low molecular weight heparin). Further studies are required to evaluate the benefits and risks of these therapies in MIS-C in neonates. Pending further studies, we recommend the following approach to MIS-C in neonates. 1. Infants with moderate to severe MIS-N may benefit from systemic glucocorticoid therapy. Methylprednisolone or prednisolone are commonly used. 2. Intravenous immunoglobulin (IVIG) is indicated in severe MIS-N requiring ICU care with cardiovascular involvement plus at least 1 other system involvement (cardiovascular involvement defined by: shock, left ventricular dysfunction, coronary artery abnormality, severe conduction abnormality, significant troponin elevation, new valvular regurgitation). Presence of coronary or peripheral aneurysms is also an indication for IVIG. Infants who meet criteria for Kawasaki disease should also receive IVIG. [23] Caution should be exercised during IVIG among neonates due to the potential risk of necrotizing enterocolitis. [24] 3. Anticoagulants: Children with MIS-N can present with vasculitis and thrombosis. [25] The incidence of thrombosis is higher in MIS-C in older children than infants and young children. [25] While low-dose aspirin, unfractionated heparin or enoxaparin are recommended in children with MIS-C, its routine use is not recommended in neonates, especially preterm infants at risk for intraventricular hemorrhage (IVH). In term infants at risk of thrombosis, and those with central lines, low-dose aspirin should be considered. Critically ill infants, admitted to the ICU with MIS-N with signs of thrombosis may benefit from prophylactic enoxaparin or unfractionated heparin. Close monitoring of PT, PTT, fibrinogen and D-dimer is necessary during anticoagulation. 4. Tocilizumab (an anti-IL-6 receptor antibody) has been used in children with MIS-C. There is no experience with the use of this therapy in neonates.
**Note**: During the neonatal period, MIS-N is relatively rare. More common causes for cardiac dysfunction and elevated Troponin or BNP such as perinatal asphyxia should be considered. The use of glucocorticoids and IVIG should be limited to indications outlined above.

Abbreviations are same as in Table 3.
